# Pancreatitis Following Biliary Endoscopic Retrograde Cholangiopancreatography for Common Bile Duct Stones in Japan: A Multicenter Prospective Cohort Study

**DOI:** 10.1002/deo2.70265

**Published:** 2025-12-23

**Authors:** Toru Maruo, Koichi Fujita, Shujiro Yazumi, Hiroko Nebiki, Kazuya Matsumoto, Mamoru Takenaka, Toshiharu Ueki, Takashi Kawamura, Hirofumi Kawamoto

**Affiliations:** ^1^ Department of Gastroenterology Fukuoka University Chikushi Hospital Chikushino Japan; ^2^ Department of Gastroenterology and Hepatology Yodogawa Christian Hospital Osaka Japan; ^3^ First Research Department Tazuke Kofukai Medical Research Institute Osaka Japan; ^4^ Department of Gastroenterology Osaka City General Hospital Osaka Japan; ^5^ Department of Multidisciplinary Internal Medicine, Division of Gastroenterology and Nephrology, Faculty of Medicine Tottori University Tottori Japan; ^6^ Department of Gastroenterology and Hepatology Kindai University Higashiosaka Japan; ^7^ Department of Preventive Services Kyoto University School of Public Health Kyoto Japan

**Keywords:** choledocholithiasis, endoscopic retrograde cholangiopancreatography, common bile duct stones, post‐ERCP pancreatitis, prophylactic pancreatic stenting

## Abstract

**Objectives:**

Post‐endoscopic retrograde cholangiopancreatography (ERCP) pancreatitis (PEP) is an adverse event of ERCP‐related procedures. The present study aimed to evaluate the incidence of PEP in patients with an intact papilla who underwent ERCP for common bile duct stones and identify risk factors and preventive measures for PEP.

**Methods:**

Between April 2017 and March 2018, ERCP‐related procedures were performed in 16,032 patients with an intact papilla at 36 institutions affiliated with the Bilio‐pancreatic Study Group of West Japan. Of these, 3739 were prospectively enrolled to investigate adverse events of ERCP‐related procedures targeting the biliary tract; the present study included 2106 patients with common bile duct stones.

**Results:**

PEP occurred in 132 patients (6.3%), and its severity was graded as mild (*n* = 104), moderate (*n* = 17), and severe (*n* = 11). Multivariable analysis identified the followin significant risk factors for PEP: age <50 years, female sex, wire‐guided biliary cannulation, pancreatic guidewire‐assisted biliary cannulation, biliary cannulation attempt duration ≥10 min, and total procedure time ≥60 min. Only pancreatic stenting was a preventive factor for PEP.

**Conclusions:**

To prevent PEP, it is necessary to avoid guidewire insertion into the pancreatic duct, prolonged biliary cannulation attempts, and long ERCP procedure time, regardless of biliary cannulation with or without guidewire assistance. When a guidewire is inserted into the pancreatic duct in patients at high risk of PEP, pancreatic stenting should be considered.

## 1 Introduction

Endoscopic retrograde cholangiopancreatography (ERCP) plays an important role in the diagnosis and treatment of biliary and pancreatic diseases. However, it is associated with adverse events, including procedural accidents, such as pancreatitis, bleeding, perforation, and infection. The incidence of ERCP‐related adverse events is 2.5%–14% [1, 2]. Among them, pancreatitis is the most common, with a reported incidence of 10.2%–14.1% [3]. In ERCP‐related procedures, the biliary orifice is separated from the pancreatic orifice in the duodenal papilla after sphincterotomy, which facilitates selective cannulation into the bile duct. As a result, the incidence of post‐endoscopic retrograde cholangiopancreatography (ERCP) pancreatitis PEP becomes lower. Thus, the incidence of ERCP‐related PEP greatly varies depending on whether the papilla is intact. Furthermore, contrast injection into the pancreatic duct may occur inadvertently during biliary ERCP, whereas it is necessary during pancreatic ERCP. Pancreatic stenting is thus a prophylactic procedure in biliary ERCP but a therapeutic procedure in pancreatic ERCP. Analysis of data without differentiating between biliary and pancreatic ERCP procedures may yield inappropriate results. Therefore, risk factors for adverse events due to ERCP need to be analyzed and discussed separately for biliary and pancreatic ERCP procedures.

We had previously established a multicenter, prospective database for biliary ERCP‐related procedures and reported particularly on the risk factors and preventive measures for PEP [4, 5, 6].

Biliary ERCP is most frequently performed to treat common bile duct stones. In recent years, the prevalence of gallbladder stones is estimated to be increasing with the increasing obese population [7], and cases of ERCP‐related procedures for common bile duct stones are expected to increase further in the future.

Therefore, we aimed to identify the factors related to PEP onset after ERCP‐related procedures for common bile duct stones in patients with an intact papilla.

## 2 Methods

### 2.1 Participants

The present study was conducted using a multicenter prospective cohort design. Between April 2017 and March 2018, ERCP‐related procedures were performed in a total of 16,032 patients with an intact papilla at 36 institutions affiliated with the Bilio‐pancreatic Study Group of West Japan. These comprised institutions with pancreatobiliary endoscopists, such as general acute‐care hospitals (*n* = 20), university hospitals (*n* = 14), and cancer centers (*n* = 2). A total of 3739 patients who underwent biliary ERCP were consecutively registered according to the protocol of the prospective registry. Of them, 2106 patients underwent ERCP‐related procedures for common bile duct stones. The study excluded patients with a diagnosis of acute pancreatitis before ERCP; patients with a postoperative reconstructed intestinal tract; patients with a history of pancreatic surgery; and patients with a severe life‐threatening systemic disease graded as American Society of Anesthesiologists Physical Status of ≥4. Serum bilirubin levels measured within 48 h before ERCP were used for the analysis. Cases that underwent endoscopic papillary large balloon dilation (EPLBD) alone and those that underwent EPLBD in addition to EST were classified into the EPLBD group. Temporary guidewire insertion into the pancreatic duct was defined as unintentional and transient passage of a guidewire into the pancreatic duct, even for a short duration, without the intention of pancreatic guidewire (PGW)‐assisted cannulation. Although various devices can be used for stone extraction and may influence total procedure time or adverse event rates, the present study included only patients with a naïve papilla undergoing their first ERCP; therefore, peroral cholangioscopy‐guided electrohydraulic lithotripsy was not included in the database. The enrolled ERCP patients received standard intravenous hydration (60–100 mL/h) until the morning after the procedure, and blood tests were performed on the day after ERCP to screen for PEP and other adverse events. The diagnostic criteria for PEP were the onset of abdominal pain persisting for ≥24 h after ERCP, exacerbation of abdominal pain, and a post‐ERCP increase in serum amylase levels to ≥3 times the upper limit of the reference value [8, 9, 10]. The American Society for Gastrointestinal Endoscopy severity grading system was used to determine the severity of PEP, bleeding, perforation, and infection, which are procedural accidents of ERCP‐related procedures [11].

### 2.2 Exposure Factors

Data were collected on patient‐related factors, procedure‐related factors, operator‐related factors, and preventive measures. The patient‐related factors included age, sex, previous PEP, and serum bilirubin level of <3 mg/dL, extrahepatic bile duct diameter of <10 mm, sphincter of Oddi dysfunction (SOD), acute cholangitis, and periampullary diverticulum. The operator‐related factors included an operator with limited experience in ERCP and a low‐volume center. The procedure‐related factors included successful deep bile duct cannulation, successful biliary cannulation, wire‐guided cannulation, PGW‐assisted biliary cannulation, precut sphincterotomy, endoscopic sphincterotomy (EST), small EST incision, endoscopic papillary balloon dilation (EPBD), EPLBD, pancreatography, temporary guidewire insertion into the pancreatic duct, intraductal ultrasonography, endoscopic biliary stenting (EBS), endoscopic nasobiliary drainage (ENBD), endoscopic mechanical lithotripsy, endoscopic biliary stone extraction combined with stone extraction with balloon or basket catheters, biliary cannulation attempt duration of ≥10 min, and total procedure time of ≥60 min. The preventive measures included pancreatic stenting, protease inhibitors, rectal administration of non‐steroidal anti‐inflammatory drugs (NSAIDs) immediately before or after ERCP, epinephrine spraying onto the papilla, and use of hypertonic contrast agents.

The intact papilla was defined as a papilla that has not been treated with EST, EPBD, EPLBD, precut sphincterotomy, endoscopic treatment of the pancreatic or biliary tract, or other procedures. Acute cholangitis was diagnosed based on systemic inflammation, cholestasis, and imaging according to the 2013 Tokyo Guidelines [12]. Operators who had performed <200 ERCP procedures or were currently performing <40 ERCP procedures per year were regarded as trainees [13]. A low‐volume center was defined as an institution where ERCP was performed in <400 cases per year. In addition, wire‐guided cannulation was defined as a procedure that was performed with guidewire assistance at the first attempt of biliary cannulation according to the policy.

### 2.3 Data Collection

A common case report form (CRF) was prepared for the present study. Data were prospectively accumulated at each institution, and clinical data recorded on CRFs were integrated by the study group. Eligibility for the study was determined before ERCP was performed, and data on ERCP‐related procedures were recorded immediately after completion of ERCP. The onset of adverse events was assessed on the day after ERCP and 1 week later, and the patient outcomes were assessed 1 month later. Data on serum bilirubin levels and extrahepatic bile duct diameters were collected in numerical form.

### 2.4 Statistical Analysis

Using commonly accepted pathological thresholds, a serum bilirubin level of 3 mg/dL and a bile duct diameter of 10 mm were used as cutoff values to divide patients into two groups for statistical analysis. Univariate analysis using the chi‐square test was performed on each factor. In addition to factors with a *p*‐value ≤0.1. Multivariable logistic regression analysis was performed on these factors, and the odds ratios (ORs) and their 95% confidence intervals (CIs) were calculated. Factors were considered to show significant differences when the *p*‐value was <0.05. Missing data were complemented with the most frequent variable for categorical data and mean or median values for continuous data. The missing rate for each variable was less than 3%. IBM SPSS Statistics Version 22 was used for statistical analyses.

## 3 Results

### 3.1 Incidence and Severity of Adverse Events of ERCP‐related Procedures

The mean age was 73.3 ± 13.7 years with 1164 men and 944 women (Table 1). One‐month outcomes were obtained from all study patients. For biliary ERCP performed in patients with common bile duct stones with an intact papilla, the success rate of biliary cannulation was 97.6%. PEP occurred in 132 patients (6.3%), and its severity was graded as mild (*n* = 104), moderate (*n* = 17), and severe (*n* = 11). As for adverse events other than PEP, bleeding occurred in 10 patients (0.47%); its severity was graded as mild (*n* = 7) and moderate (*n* = 3), whereas no severe bleeding was reported in any patient. Perforation occurred in 12 patients (0.57%); its severity was graded as mild (*n* = 5), moderate (*n* = 4), severe (*n* = 2), and fatal (*n* = 1). Infections occurred in 15 patients (0.71%), and the severity was graded as mild (*n* = 11) and moderate (*n* = 4); no severe infection was found in any patient. The observed infections included cholangitis (*n* = 7), cholecystitis (*n* = 6), and others (*n* = 2).

**TABLE 1:** Baseline clinical characteristics of the study patients.

Patients	*n*	2106
Sex	Male/female, *n*	1164/942
Age	Years, mean (SD)	73.3 (13.7)
	< 30, *n* (%)	22 (1.0)
	30–49, *n* (%)	115 (5.5)
	50–69, *n* (%)	558 (26.5)
	70–89, *n* (%)	1260 (59.8)
	≥ 90, *n* (%)	151 (7.2)
ASA‐PS	ASA 1/2/3, *n*	1027/768/311

Abbreviations: ASA‐PS, American Society of Anesthesiologists Physical Status; SD, standard deviation

### 3.2 Univariable Analysis of Exposure Factors for PEP

Univariable analysis revealed the following 13 factors with a *p*‐value of <0.1, which were selected as candidate factors for the development of PEP: young age of <50 years (*p* = 0.044), female sex (*p* = 0.003), serum bilirubin level of <3 mg/dL (*p* = 0.03), and acute cholangitis (*p* = 0.009) among patient‐related factors; wire‐guided cannulation (*p* = 0.031), PGW‐assisted biliary cannulation (*p* < 0.001), precut sphincterotomy (*p* = 0.006), pancreatic contrast injection (*p* < 0,001), temporary guidewire insertion into the pancreatic duct (*p* = 0.03), biliary cannulation attempt duration of ≥10 min (*p* < 0.001), and total procedure time of ≥60 min (*p* < 0.001) among procedure‐related factors; and pancreatic stenting (*p* = 0.013) and rectal administration of NSAIDs (p = 0.03) among preventive measures. The trainee status and the low‐volume center, which are operator‐related factors, were not found to be candidate factors for PEP development (Table 2).

**TABLE 2:** Prognostic factors for PEP by univariable and multivariable analysis.

Factors	n	PEP (%)	Univariable analysis		Multivariable analysis	
			OR (95% CI)	*p*‐value	OR (95% CI)	p‐value
**Patient‐related factors**						
Age < 50 years	137	14 (10.22)	1.78 (0.96‐3.19)	0.04	2.24 (1.20‐4.18)	0.02
Female sex	942	76 (8.06)	1.73 (1.21‐2.47)	0.01	1.65 (1.13‐2.39)	0.009
SOD	12	1 (8.33)	0.75 (0.52‐1.07)	0.12		
Previous pancreatitis	43	5 (11.63)	1.02 (0.99‐1.06)	0.19		
Serum bilirubin <3 mg/dL	1566	109 (6.96)	1.65 (1.04‐2.62)	0.03	1.29 (0.78‐2.13)	0.329
Extrahepatic bile duct diameter <10 mm	1018	71 (6.97)	1.26 (0.88‐1.79)	0.21		
Acute cholangitis	948	45 (4.75)	0.61 (0.42‐0.89)	0.01	0.71 (0.45‐1.05)	0.088
Periampullary diverticulum	683	36 (5.27)	0.77 (0.52‐1.14)	0.21		
**Operator‐related factors**						
Trainee	1063	67 (6.31)	1.01 (0.71‐1.44)	0.96		
Institution size <400 beds	451	24 (5.32)	0.81 (0.51‐1.27)	0.38		
**Procedure‐related factors**						
Successful deep bile duct cannulation	2053	127 (6.19)	0.59 (0.23‐1.52)	0.24		
Unsuccessful biliary cannulation	50	5 (10)	1.69 (0.66‐4.32)	0.24		
Wire‐guided cannulation	474	40 (8.44)	1.54 (1.05‐2.27)	0.03	1.86 (1.23‐2.82)	0.003
PGW‐assisted biliary cannulation	448	69 (15.43)	4.60 (3.21‐6.59)	<0.001	2.79 (1.72‐4.54)	<0.001
Precut sphincterotomy	105	14 (13.38)	2.45 (1.36‐4.43)	0.01	0.86 (0.44‐1.69)	0.67
EST	1602	93 (5.82)	0.73 (0.49‐1.08)	0.11		
Small EST incision	1059	59 (5.57)	1.21 (0.78‐1.57)	0.25		
EPBD	129	10 (7.65)	1.28 (0.65‐2.49)	0.45		
EPLBD	120	4 (3.34)	0.50 (0.18‐1.38)	0.24		
Pancreatic contrast injection	734	76 (10.35)	2.71 (1.89‐3.87)	<0.001	1.30 (0.83‐2.04)	0.253
Temporary GW insertion into the pancreatic duct	183	17 (9.29)	1.61 (1.06‐2.75)	0.03		
IDUS	129	12 (9.3)	1.59 (0.85‐2.95)	0.14		
EBS	880	64 (7.27)	1.33 (0.94‐1.89)	0.12		
ENBD	255	11 (4.31)	0.64 (0.34‐1.21)	0.21		
Endoscopic biliary stone extraction	1223	69 (5.64)	0.78 (0.55‐1.10)	0.17		
Biliary cannulation attempt duration ≥10 min	621	82 (13.2)	4.36 (3.02‐6.28)	<0.001	2.19 (1.34‐3.42)	0.001
Total procedure time ≥60 min	202	37 (18.31)	4.26 (2.82‐6.44)	<0.001	2.56 (1.62‐4.07)	<0.001
**Preventive measures**						
EPS	199	21 (10.55)	1.91 (1.17‐3.11)	0.01	0.52 (0.30‐0.87)	0.018
Protease inhibitors	1651	108 (6.54)	1.25 (0.79‐1.97)	0.38		
NSAIDs	357	32 (8.96)	1.62 (1.07‐2.46)	0.03	1.21 (0.78‐1.89)	0.397
Epinephrine	642	32 (4.98)	0.71 (0.47‐1.08)	0.12		
Hypertonic contrast agents	1156	70 (6.06)	0.92 (0.65‐1.31)	0.65		

Abbreviations: CI, confidence interval; EBS, endoscopic biliary stenting; ENBD, endoscopic nasobiliary drainage; EPBD, endoscopic papillary balloon dilation; EPLBD, endoscopic papillary large balloon dilation; EPS, endoscopic pancreatic stenting; EST, endoscopic sphincterotomy; GW, guidewire; IDUS, intraductal ultrasonography; NSAIDS, nonsteroidal anti‐inflammatory drugs; OR, odds ratio; PEP, post‐endoscopic retrograde cholangiopancreatography (ERCP) pancreatitis; PGW, pancreatic guidewire; SOD, sphincter of Oddi dysfunction.

### 3.3 Multivariable Analysis of Exposure Factors for PEP

Wire‐guided cannulation and temporary guidewire insertion into the pancreatic duct were evaluated for multicollinearity using the variance inflation factor, and because a high correlation was observed between the two variables, temporary guidewire insertion into the pancreatic duct was excluded. Multivariable logistic regression analysis was then performed using the remaining 13 factors with a *p*‐value ≤ 0.1 in the univariable analysis. This analysis identified six risk factors and one protective factor for the development of PEP during biliary ERCP for common bile duct stones. The risk factors included young age of <50 years (OR: 2.13 [95% CI: 1.13–4.01]) and female sex (OR: 1.64 [95% CI: 1.12–2.39]) among the patient‐related factors and wire‐guided cannulation (OR: 1.70 [95% CI: 1.10–2.63]), PGW‐assisted biliary cannulation (OR: 4.00 [95% CI: 2.34–6.85]), temporary guidewire insertion into the pancreatic duct (OR: 2.59 [95% CI: 1.36–4.94]), biliary cannulation attempt duration of ≥10 min (OR: 1.94 [95% CI: 1.23–3.05]), and total procedure time of ≥60 min (OR: 2.91 [95% CI: 1.80–4.70]) among the procedure‐related factors. The protective factor was a preventive measure of pancreatic stenting (OR: 0.51 [95% CI: 0.29–0.89]). Moreover, patients with acute cholangitis (OR: 0.70 [95% CI: 0.46‐1.06]) were less likely to develop PEP (Table 2).

## 4 Discussion

Young age [14] and female sex [15] are known risk factors for the development of PEP due to ERCP‐related procedures, including biliary and pancreatic procedures. Multivariable analysis identified young age and female sex as risk factors among the patient‐related factors, and this was consistent with previously reported results. Among the procedure‐related factors, wire‐guided cannulation, temporary guidewire insertion into the pancreatic duct, biliary cannulation attempt duration of ≥10 min, and total procedure time of ≥60 min were significant risk factors for PEP development.

Contrast injection and guidewire insertion into the pancreatic duct have previously been reported as definite risk factors for PEP due to ERCP [15, 16, 17]. Additionally, PGW‐assisted biliary cannulation has been reported as a risk factor for PEP [18]. Most patients who undergo ERCP‐related procedures for common bile duct stones have a normal pancreas, of which the function can be assumed to be well preserved. A guidewire was temporarily inserted into the pancreatic duct when biliary cannulation was difficult. This suggests that PEP onset may be attributed to damage of the pancreatic duct due to mechanical stimulation of the main or branch pancreatic duct by the guidewire.

Some studies report that biliary cannulation performed using wire‐guided cannulation reduces the incidence of ERCP‐related PEP [19, 20], whereas other studies report that the incidence of PEP remains the same [17, 21, 22]. However, the present study revealed that wire‐guided cannulation performed to achieve biliary cannulation was a risk factor for PEP development. Biliary cannulation was first attempted using wire‐guided cannulation in 474 patients, of whom 40 (8.4%) developed PEP. The bile duct was successfully cannulated using wire‐guided cannulation in 296 patients (62.4%), and a guidewire was inserted into the pancreatic duct in 178 patients (37.6%) because of difficult biliary cannulation. Among these patients, contrast was injected into the pancreatic duct in 86 patients, and the guidewire was inserted into the pancreatic duct without injection in 92 patients. Although wire‐guided cannulation was first attempted, it was ultimately converted into PGW‐assisted biliary cannulation in 101 patients (21.3%) because of difficulty performing biliary cannulation. Univariable analysis on factors related to wire‐guided cannulation and PEP revealed that contrast injection into the pancreatic duct was infrequent during wire‐guided cannulation; that wire‐guided cannulation was poorly associated with the rate of conversion to PGW‐assisted biliary cannulation, biliary cannulation attempt duration, and the success rate of biliary cannulation; and that temporary guidewire insertion into the pancreatic duct was common during wire‐guided cannulation (Table 2). Although pancreatic contrast injection was rare during wire‐guided cannulation, wire‐guided cannulation was a risk factor for PEP because a guidewire was often inserted into the pancreatic duct. Many patients had a normal pancreas, which may be more susceptible to inflammation even with minor stimulation; this may reflect the risk of pancreatic duct or papillary injury caused by guidewire manipulation.

The incidence of PEP increases as the biliary cannulation attempt duration or procedure time is prolonged [23, 24, 25], and biliary cannulation attempt duration is associated with PEP severity [26]. The present study revealed that biliary cannulation attempt duration of ≥10 min and total procedure time of ≥60 min were risk factors for PEP. These findings may reflect the difficulty in performing biliary cannulation or the performance of complex procedures after biliary cannulation. In addition, previous studies have indicated that pancreatic stenting is an effective preventive measure against PEP [27, 28]. In the present study, pancreatic stenting increased the risk of PEP in univariable analysis but was a preventive factor for PEP in multivariable analysis. A guidewire must be inserted into the pancreatic duct for pancreatic stent placement. Guidewire insertion into the pancreatic duct is a strong risk factor for PEP; however, among those cases, many patients received pancreatic stent placement, which likely prevented PEP. Therefore, the intrinsic preventive effect of pancreatic stenting may have emerged and acted as an independent protective factor. ERCP for common bile duct stones increases the risk of developing PEP in patients without acute cholangitis [29]. The present study showed a lower incidence of PEP in patients with cholangitis who underwent ERCP for common bile duct stones. In this study, NSAIDs did not show a preventive effect against PEP; however, the timing and dose of administration varied considerably among institutions, with many cases receiving NSAIDs after rather than before ERCP, and dose reduction or avoidance in elderly patients or those with renal impairment. Such heterogeneity may have masked the true preventive effect of NSAIDs.

The major strengths of the present study include its prospective, large‐scale, multicenter design involving as many as 36 institutions, the sample size of 2106 patients with an intact papilla who underwent ERCP‐related procedures for common bile duct stones, and the large data directly connected to daily clinical practice.

The limitations of the study are the observational study design based on actual clinical practice and the inclusion of many patients with common bile duct stones undetectable by imaging or with acute cholangitis due to biliary sludge. Although stone size and number could influence the duration of stone extraction, in the present study, the number of common bile duct stones, stone diameter, and degree of pancreatic atrophy due to chronic pancreatitis were not included as factors because the data were collected to examine procedural accidents of biliary ERCP, particularly PEP, instead of the success of biliary cannulation and stone extraction. Further research is warranted to identify patient‐ or procedure‐related factors associated with adverse events and long‐term recurrence, which may help in the development of individualized treatment strategies. Future studies should incorporate stone number and size into the analysis.

In conclusion, the incidence of PEP was 6.3% in patients with an intact papilla who underwent biliary ERCP for common bile duct stones. To prevent PEP, it is necessary to avoid guidewire insertion into the pancreatic duct, prolonged biliary cannulation attempts, and long ERCP procedure time, regardless of biliary cannulation with or without guidewire assistance. When a guidewire is inserted into the pancreatic duct in patients at high risk of PEP, pancreatic stenting should be considered.

## Author Contributions

**Conceptualization**: Toru Maruo, Koichi Fujita, Shujiro Yazumi, Kazuya Matsumoto, Mamoru Takenaka, Toshiharu Ueki, and Hirofumi Kawamoto. **Data curation**: Toru Maruo, Koichi Fujita, Shujiro Yazumi, Kazuya Matsumoto, Mamoru Takenaka, and Hirofumi Kawamoto. **Formal analysis**: Toru Maruo, Koichi Fujita, and Takashi Kawamura. **Investigation**: Toru Maruo, Koichi Fujita, Shujiro Yazumi, Hiroko Nebiki, Kazuya Matsumoto, Mamoru Takenaka, Toshiharu Ueki, and Hirofumi Kawamoto. **Methodology**: Toru Maruo, Koichi Fujita, Shujiro Yazumi, Kazuya Matsumoto, Mamoru Takenaka, Toshiharu Ueki, and Hirofumi Kawamoto. **Project administration**: Shujiro Yazumi and Hirofumi Kawamoto. Supervision: Koichi Fujita, Shujiro Yazumi, Toshiharu Ueki, Takashi Kawamura, and Hirofumi Kawamoto. **Writing – original draft**: Toru Maruo. **Writing – review editing**: Toru Maruo.

## Funding

This research received no specific grant from any funding agency in the public, commercial, or not‐for‐profit sectors.

## Ethics Statement

The present study was conducted after its outline was registered in the University Hospital Medical Information Network Clinical Trial Registry (UMIN000024820). It was a non‐invasive cohort study, and no tissue samples were collected. The outline of the study was posted on the website and bulletin board of each institution to inform the participants and provide them with an opportunity to decline participation. The study was approved by the institutional review board of each institution.

## Conflicts of Interest

The authors declare no conflict of interest.
